# Phenotypic and proteomic approaches of the response to iron-limited condition in *Staphylococcus lugdunensis*

**DOI:** 10.1186/s12866-020-02016-x

**Published:** 2020-10-28

**Authors:** Marion Aubourg, Anne Dhalluin, François Gravey, Marine Pottier, Nicolas Thomy, Benoit Bernay, Didier Goux, Matthieu Martineau, Jean-Christophe Giard

**Affiliations:** 1 Université de Caen Normandie, EA4655 U2RM (équipe «Antibio-résistance»), CHU de Caen, Caen, France; 2 Université de Caen Normandie, GRAM 2.0, CHU de Caen, Service de Microbiologie, Caen, France; 3 Plateforme Proteogen SFR ICORE 4206, Université de Caen Normandie, Caen, France; 4 Centre de Microscopie Appliquée à la Biologie, Université de Caen Normandie IFR ICORE, Caen, France

**Keywords:** *Staphylococcus lugdunensis*, Iron limitation, Proteomic, Virulence, Oxydative stress

## Abstract

**Background:**

*Staphylococcus lugdunensis* is a coagulase-negative *Staphylococcus* part of the commensal skin flora but emerge as an important opportunistic pathogen. Because iron limitation is a crucial stress during infectious process, we performed phenotypic study and compared proteomic profiles of this species incubated in absence and in presence of the iron chelator 2,2′-dipyridyl (DIP).

**Results:**

No modification of cell morphology nor cell wall thickness were observed in presence of DIP. However iron-limitation condition promoted biofilm formation and reduced the ability to cope with oxidative stress (1 mM H_2_O_2_). In addition, *S. lugdunensis* N920143 cultured with DIP was significantly less virulent in the larvae of *Galleria mellonella* model of infection than that grown under standard conditions. We verified that these phenotypes were due to an iron limitation by complementation experiments with FeSO_4_. By mass spectrometry after trypsin digestion, we characterized the first iron-limitation stress proteome in *S. lugdunensis*. Among 1426 proteins identified, 349 polypeptides were differentially expressed. 222 were more and 127 less abundant in *S. lugdunensis* incubated in iron-limitation condition, and by RT-qPCR, some of the corresponding genes have been shown to be transcriptionally regulated. Our data revealed that proteins involved in iron metabolism and carriers were over-expressed, as well as several ABC transporters and polypeptides linked to cell wall metabolism. Conversely, enzymes playing a role in the oxidative stress response (especially catalase) were repressed.

**Conclusions:**

This phenotypic and global proteomic study allowed characterization of the response of *S. lugdunensis* to iron-limitation. We showed that iron-limitation promoted biofilm formation, but decrease the oxidative stress resistance that may, at least in part, explained the reduced virulence of *S. lugdunensis* observed under low iron condition.

**Supplementary information:**

**Supplementary information** accompanies this paper at 10.1186/s12866-020-02016-x.

## Background

Described for the first time in 1988, *Staphylococcus lugdunensis* is a coagulase-negative *Staphylococcus* (CoNS) that is part of the normal human skin flora but can cause serious infections similar to those generated by *Staphylococcus aureus* [1, 2]. Recognized as an emerging opportunistic pathogen, *S. lugdunensis* is responsible of acute endocarditis, skin and soft tissue infections, brain abscesses and osteoarticular infections [3]. Some virulence and opportunistic determinants have been identified mainly based on whole-genome analyses and by comparison with other Staphylococci but few of them have been experimentally characterized [4]. Unlike other CoNS such as *Staphylococcus epidermidis*, this bacterium is less frequently retrieved among clinical samples but has a high level of virulence that makes it more similar to *S. aureus* [3]. Notably, *S. lugdunensis* is the only CoNS that has an iron-regulated surface determinant (Isd) system for iron capture and metabolism close to that of *S. aureus* [5]. This Isd system allows binding of hemoglobin with subsequent removal of heme that is transported into the bacterial cytoplasm. Heme is then degraded and nutrient iron is released [2]. In these two Staphylococci species, the *isd* operon is expressed under iron deficiency conditions allowing bacteria to overcome this deficiency encountered during colonization of the host and invasive diseases [6, 7]. During infection, iron acquisition appears essential for survival and spread of pathogens. In *S. aureus*, the use of heme as an iron source through its Isd system contributes to its full virulence but this has not been studied in *S. lugdunensis* yet [8].

However, the ability to adapt and to cope with stresses are important for the virulence of opportunistic pathogens. In response to environmental stimuli, regulatory cascades leads to a fine-tuning of metabolic and virulence genes expression [9, 10]. Among Omics technologies, proteomics occupies a strategic place because it deals with enzymes which are the true effectors of the cellular physiology. Compared to genomic sequences that provide the gene content, proteomic is dedicated to the identification of and quantification of polypeptides occurring under specific environmental conditions. For Staphylococci, this tool has been used to address physiological and pathophysiological questions and allowed to bring comprehensive understanding of stress response, cell physiology, host-pathogen interactions, regulatory networks and virulence [11–13].

In this study, we performed phenotypic and comparative proteomic analysis to identify the iron-limitation proteome in *S. lugdunensis*. These data reflected the better ability to form biofilm and the increased susceptibility to H_2_O_2_ when cell were iron limited. Moreover, we showed that iron deprivation had a negative impact on the pathogenicity using the larvae of *Galleria mellonella* model of infection. Under this condition, transporters and enzymes involved in iron metabolism were over expressed whereas proteins linked to the oxidative stress response were less abundant.

## Results

### Impact of iron limitation on growth of *S. lugdunensis* N920143

**Fig. 1:**
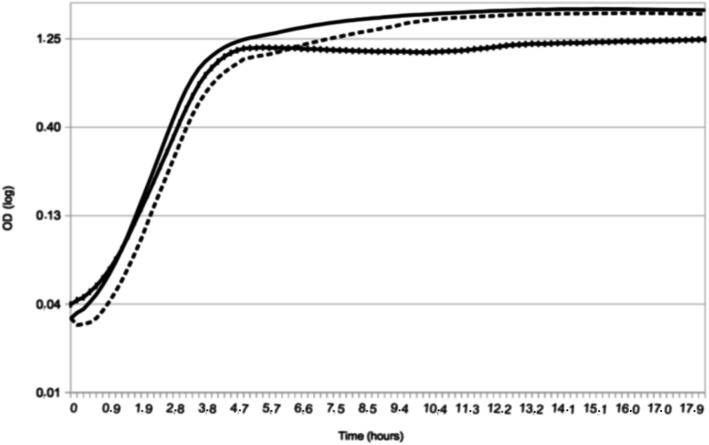
Representative growth curves of *S. lugdunensis* N920143 in BHI (continuous line), in BHI with 350 μM DIP (dashed line) and in BHI with 350 μM DIP and 2 mM FeSO_4_ (spaced dashed line)

With the aim to perform comparative proteomic analyses with cells harvested at the same point of the growth curve, we first evaluated bacterial growth under different conditions. Growth of *S. lugdunensis* N920143 was studied over 18 h. As shown in Fig. 1, optimal growth rate was very slightly reduced by iron limitation showing that 350 μM of iron chelator 2,2′-dipyridyl (DIP) was not toxic for this bacteria. However, in presence of DIP, a slightly reduced cell density in stationary phase was observed. *S. lugdunensis* cells cultivated under iron-limited condition reached a final OD of 1.3 versus 1.9 for cells cultivated in absence of iron chelator (*p* = 1 × 10^− 4^) (Fig. 1). In order to confirm that the observed phenotype was caused by iron limitation we performed complementation experiments in presence of 2 mM FeSO_4_. As expected, when cells were incubated in BHI with DIP and FeSO_4_, the final OD was similar as bacteria cultivated in BHI (Fig. 1).

### Phenotypical impacts of iron limitation

We first investigated the cell morphology of *S. lugdunensis* N920143 strain, at the onset of stationary phase (OD of 1) and after 24 h in BHI without or with 350 μM DIP. High-resolution TEM observations showed similar morphology as well as cell wall thickness (Fig. S1).

**Fig. 2:**
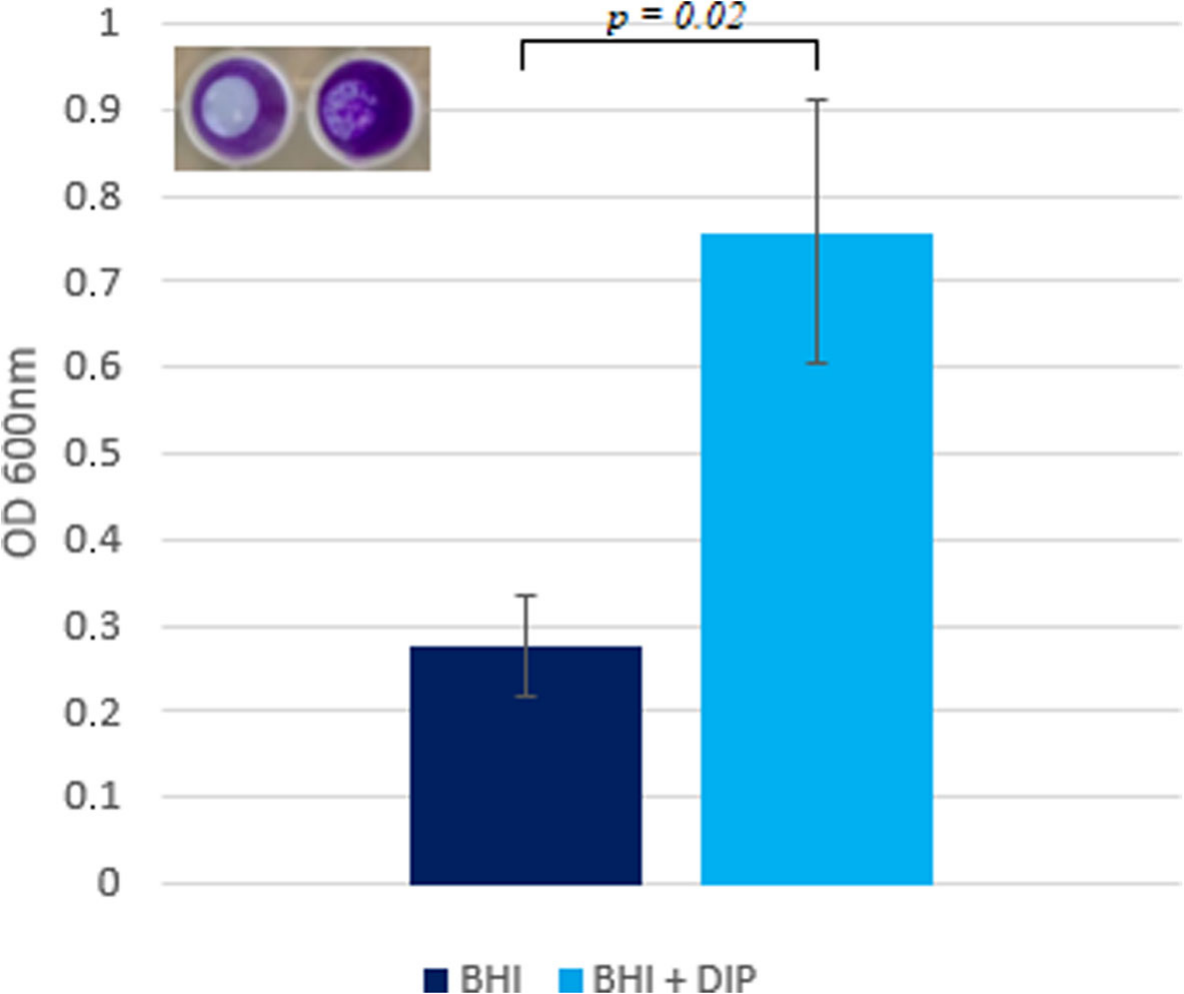
Biofilm formation by *S. lugdunensis* after 24 h of growth in BHI without (dark blue bar) or with 350 μM DIP (light blue bar). Error bars represent the standard deviations of three independent experiments. Inlay, crystal violet-stained wells (left, cells grown in BHI, right, cells grown in BHI with DIP). A slightly reduced optimal growth rate and cell density in stationary phase were observed for *S. lugdunensis* cells cultivated under iron-limited condition

We then verified that our experimental condition of iron limitation was correlated to a greater ability to produce biofilm. As shown in Fig. 2, *S. lugdunensis* grown in BHI supplemented with 350 μM DIP made significantly more biofilm than when incubated in BHI (*p =* 0.02). After 24 h of culture, the OD were of 0.75 and 0.28 for bacteria cultured in presence and in absence of DIP in BHI, respectively (Fig. 2).

**Fig. 3:**
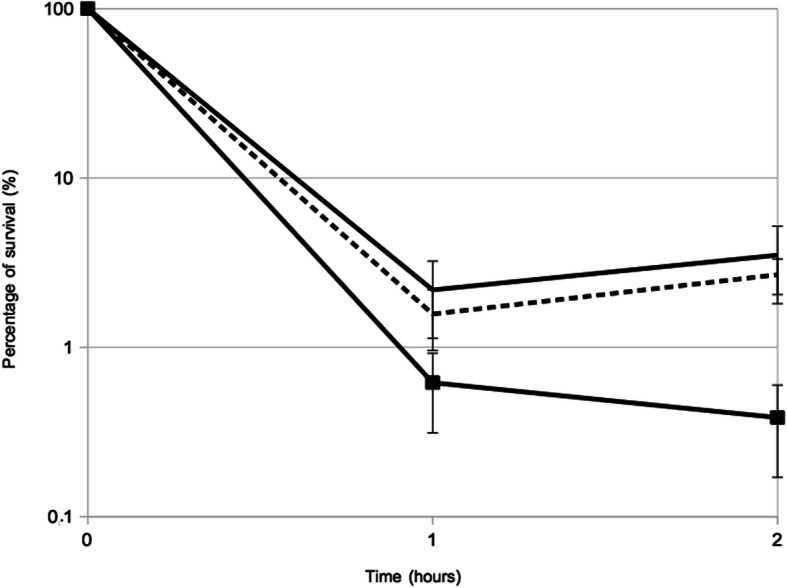
Representative survival curves at 1 mM H_2_O_2_ of *S. lugdunensis* N920143 in BHI (continuous line), in BHI with 350 μM DIP (hatched continuous line) and in BHI with 350 μM DIP and 2 mM FeSO_4_ (spaced dashed line)

In the attempt to correlate iron-limitation with the ability to cope with oxidative stress, we analyzed the survival of *S. lugdunensis* in presence of lethal dose of H_2_O_2_. As shown in Fig. 3, the oxidative stress triggered by the addition of 1 mM H_2_O_2_ had more significant effect towards cells incubated with iron chelator than bacteria incubated in BHI. After 2 h, 3.5 and 0.4% of cells were viable when incubated without and with DIP, respectively (*p* = 0.033). Moreover, addition of FeSO_4_ canceled the effect of the iron chelator (Fig. 3). We also showed that addition of sub-lethal concentration of H_2_O_2_ (0.4 mM) had much more impact on growth rate (two fold reduced) for cells grown in presence of DIP (Fig. S2).

**Fig. 4:**
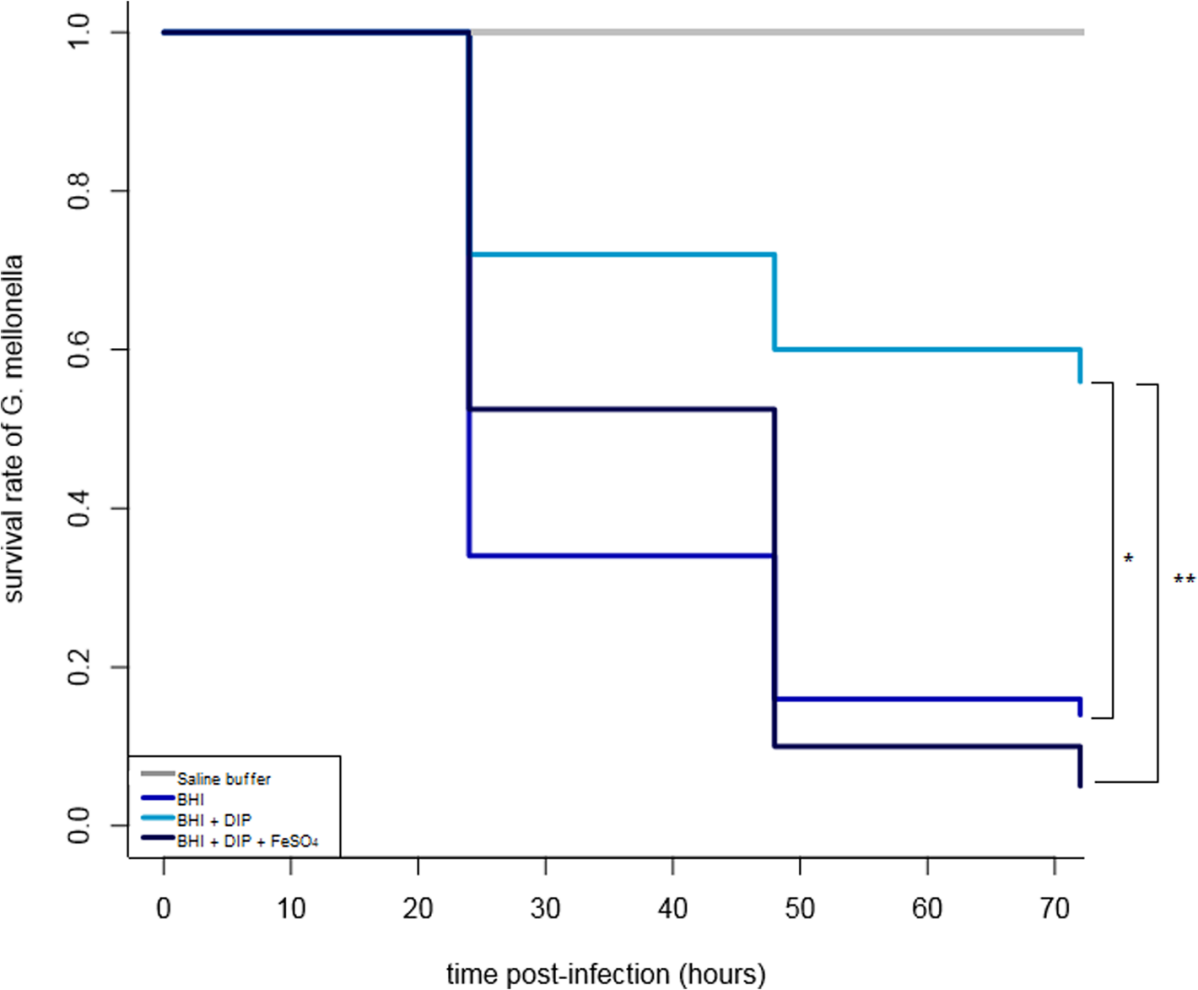
Kaplan-Meier survival curves of larvae of *G. mellonella* after infection with around 1 × 10^7^ CFU of *S. lugdunensis* previously cultivated in BHI (dark blue line), in BHI supplemented with 350 μM DIP (light blue line), in BHI with 350 μM DIP and 2 mM FeSO_4_ (black), and sterile saline buffer without cells (grey line). *: *p* = 1 × 10^− 5^. **: *p* = 3.9 × 10^− 6^. In this animal model, virulence of *S. lugdunensis* previously cultivated in presence of DIP was significantly reduced.

It was suspected that iron-limitation condition may affect the pathogenicity of *S. lugdunensis*. Thus, we evaluated the impact of pre-incubation with iron chelator on the virulence of *S. lugdunensis* N920143 using the *G. mellonella* model of infection. Infection caused by *S. lugdunensis* previously cultivated in presence of DIP was significantly less severe than that caused by cells cultivated in BHI (Fig. 4). Indeed, after 72 h post-infection, 14 and 56% of the larvae survived when infected with bacterial cells grown in BHI or in iron-limitation condition, respectively (*p =* 3.4 × 10^− 6^) (Fig. 4) Note that *S. lugdunensis* grown in BHI with DIP and FeSO_4_ displayed the same pathogenicity as cells incubated in BHI, arguing for a role of iron in these phenotypes (Fig. 4). In addition, to verify that our observations were not due to a reduced fitness of stressed cells, we inoculated iron-rich medium and iron-limited medium with bacteria previously grown in BHI or BHI with DIP. In all cases, the growth curves were similar (data not shown).

### The iron-limitation proteome

**Fig. 5:**
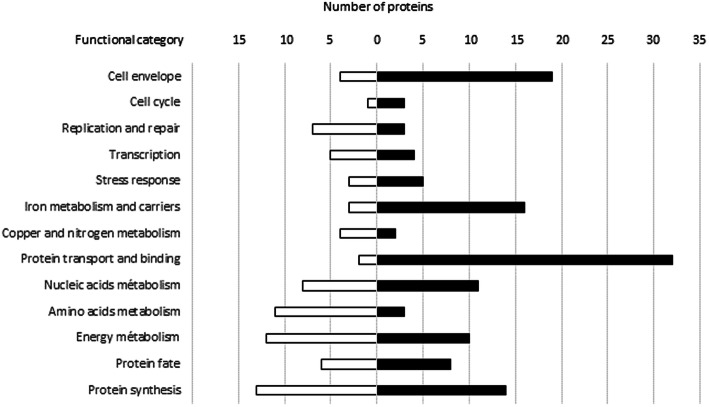
Functional classification of proteins significantly upregulated (black bars) and downregulated (white bars) of growing cell (OD of 1) of *S. lugdunensis* N920143 incubated in BHI with 350 μM DIP compared to *S. lugdunensis* N920143 in BHI

The iron limitation proteome of *S. lugdunensis* N920143 was analyzed by mass-spectrometry after trypsin digestion. Based on growth curve analysis, proteins were extracted from cells grown with or without DIP harvested at the end of the exponential phase (OD of 1). About 1426 of the 2368 proteins potentially coded by the genome have been identified and quantified using protein extracts from three biological replicates. 349 polypeptides appeared differentially expressed by *S. lugdunensis* grown in presence of DIP and were categorized according to their cellular functions (Fig. 5). Among those, 222 were significantly more and 127 less abundant in cells incubated in iron limitation condition (fold changes greater or smaller than 2 with a *p*-value less than 0.05) (Fig. 5, Table S1). 209 polypeptides could be assigned to a specific cellular pathway, and it was possible to detect proteins related to several metabolisms (nucleic acids, amino acids, energy, ions, and proteins), stress response, cell envelope, cell cycle, DNA replication and transcription (Fig. 5).

Not surprisingly, several proteins (*n* = 16) involved in the iron metabolism and carriers were over-produced. Thus, IsdJ, IsdG, IsdC and IsdE (members of Isd system) were 8.7, 8.1, 4.8 and 5.7 fold more abundant, respectively, and two siderophore ABC transporters were more than 7 fold over-expressed (Table S1). Notably, 32 transporter proteins of which a majority were of ABC type, were obviously present in higher amount in cells incubated with DIP (Fig. 5). Despite the lack of alteration in cell morphology or cell wall thickness (Fig. S1), the iron limitation stress condition appeared also effecting cell wall metabolism because nineteen polypeptides involved in these pathways were more abundant and four less (Fig. 5).

We detected one protein related to virulence (the LugC subunit of the lugdunin) that were 0.7 fold under-produced when iron lacked, suggesting a possible impact on the pathogenicity of *S. lugdunensis*. Moreover, catalase, the major enzyme involved in the oxidative stress response in Staphylococci and known as virulence factor, was one of the most repressed protein identified (3.8 fold), likely reflecting a decrease of resistance to oxidant (Fig. 5). Interestingly, the two enzymes linked to nitrate metabolism (nitrate reductase and nitrite reductase), the cognate two-component regulatory system (NreBC) as well as the nitric oxide synthase were all detected as significantly repressed (between 0.9 to 4.6 fold less abundant). SufB and SufD, two members of the iron-sulfur cluster biosynthetic system were also observed as under-represented proteins (0.5 and 0.8 fold, respectively) when cells were in iron-limitation condition (Fig. 5).

With the aim of knowing whether some of proteins differentially synthesized due to the presence of DIP were under transcriptional regulation, we performed qPCRs for genes coding for proteins showing the highest and lowest levels of abundance excluding enzyme involved in translation that are usually present in high amount into cells. Thus, expressions of genes from the *isd* locus were tested and revealed that transcription of *isdJ* and *isdB* were 183 and 240 fold induced under iron limitation condition, respectively (Table 1). In addition, expression of *katA* (encoding catalase) was more than 6.35 fold reduced corroborating proteomic data. Expression of other genes coding for proteins among the most over and under-expressed also revealed significant transcriptional induction and repression, respectively (Table 1). Of note, no transcriptional modification of genes coding for uptake regulatory elements (*fur*, *zur* and *perR*) were observed when cells were incubated with DIP (data not shown).

**Table 1:** Analysis of transcriptional level of selected genes coding for proteins up and down-regulated in *S. lugdunensis* cultivated in BHI with DIP versus grown in BHI

Protein	Gene	Proteomic fold-change^a^	Transcriptomic fold-change	Transcriptomic ***p***-value
Iron-regulated Surface Determinant J	*isdJ*	8.74	183.64	7.50e-04
Iron-regulated Surface Determinant B	*isdB*	8.18	240.73	3.19e-05
Iron-siderophore ABC transporter	*sirA*	7.31	21.24	3.40e-03
Transferin-binding protein	*SLUG_14730*	7.08	45.84	1.78e-05
Siderophore ABC transporter	*sstD*	7.06	95.74	4.02e-05
Catalase	*katA*	-3.83	-6.35	6e-04
Nitrate reductase subunit alpha	*narG*	-4.65	-1.96	3.81e-02
Formate dehydrogenase subunit alpha	*SLUG_07190*	-5.04	-2.23	7.24e-03
NAD(P)/FAD-dependent oxidoreductase	*nasD*	-6.04	-2.21	1.12e-02

^a^from Table S1

## Discussion

*S. lugdunensis* most often behaves more like the coagulase-positive *S. aureus* than other CoNS by its apparently high virulence [3]. It can cause various types of infections, ranging from localized to systemic diseases [3]. Although *S. lugdunensis* is recognized as an important pathogen, few studies have been conducted to determine mechanism of its pathogenicity and to identify virulence factors. Iron acquisition has been described as important for the full virulence of *S. aureus* and *S. lugdunensis* is the only CoNS that owns an Isd system responsible for the acquisition of iron from hemoglobin and heme in vivo [5, 7]. In order to analyze the global cellular response triggered by iron limitation, we characterized proteomes of *S. lugdunensis* cells grown in BHI with and without DIP. First, we showed that growth rate and cell morphology were not affected by addition of an iron chelator (DIP) but we observed a reduced cell density in stationary phase. Comparative proteomic study showed that 349 proteins were differentially expressed in presence of DIP out of 1400 polypeptides identified. Several metabolisms were impacted by this condition and enzymes involved in transcriptional regulation (3 more abundant and 5 less) were identified suggesting a complex regulatory network. Our transcriptomic results performed on genes coding for some proteins among those mostly over and under-expressed under iron-limitation condition, suggested that this deregulation mainly occurred at the transcriptional level.

As expected, several proteins involved in iron metabolism and capture were in greater amount when cells were incubated with DIP. Among them, we found members of the *isd* system, siderophores, as well as membrane associated proteins and ABC transporters. We confirmed that the better ability of *S. lugdunensis* to form biofilm under iron-restricted condition was associated with the induction of Isd proteins, correlated to a spectacular increase of transcription of *isd* genes. Missineo and collaborators showed an iron-concentration dependence of IsdC production and biofilm formation in *S. lugdunensis* with a critical concentrations of 1 to 50 μM of FeCl_3_ [14]. IsdB captures heme from hemoglobin and transfers it to IsdC located into the peptidoglycan. Heme is then transferred to the membrane located transporter IsdEF and then broken by oxygenases IsdG and IsdI leading to releasing free iron in the cytoplasm [6]. As already published, an *isdC* mutant of *S. lugdunensis* was defective in biofilm production due to its role in cells attachment and cell-cell interactions [14]. Nevertheless, the over-production of several transporters likely played also in favor of the increase in biofilm formation by rising the production of extra-cellular compounds [15].

Interestingly, our proteomic analysis showed that the iron limitation condition was also linked to the oxidative stress response in *S. lugdunensis*. Repression of catalase (KatA) (at proteomic and transcriptomic levels) was observed when cells were incubated with DIP and these results were phenotypically validated by a higher susceptibility to H_2_O_2_. In presence of free iron, H_2_O_2_ can undergo Fenton’s reaction leading to the formation of very deleterious hydroxyl radicals [16]. Under iron limitation condition, it appeared that hydrogen peroxide molecules were able of damaging bacterial cells. In bacteria, peroxidase activities are important to escape the host oxidative defenses during the immune response against infectious processes [17]. In *S. aureus*, KatA was correlated with virulence in clinical isolates and appeared required for nasal colonization and may be then qualified as a virulent factor [18, 19]. We also showed repression of SufB and SufD enzymes from the SufCDSUB system involved in synthesize of inorganic iron-sulfur (Fe-S) cluster prosthetic groups. In *S. aureus*, Roberts and co-workers revealed that decreased Suf function resulted in global metabolic defects, sensitivity to reactive oxygen species, and in decreased survival in human polymorphonuclear neutrophils (PMNs) [20]. Among the significantly repressed proteins observed we also found the nitrate reductase, nitrite reductase and the cognate two-component regulatory system (NreBC). These enzymes are involved in degradation of reactive nitrogen species (RNS) allowing bacterial cells to cope with nitrosative stress encountered into phagocytic cells [17]. Moreover, the nitric oxide synthase, under-expressed under iron limitation condition in *S. lugdunensis*, has been shown to protect *S. aureus* against killing by neutrophils, as well as being involved in abscess generation in a mouse subcutaneous infection model [21, 22]. Of note, the decrease of enzymes involved in nitrate/nitrite degradation under low-iron condition may also plays in favor of biofilm formation in *S. lugdunensis* since nitrite (either as product of respiratory nitrate reduction or experimental addition) inhibited *S. aureus* biofilm formation [23]. LugC that is part of the enzymatic complex for the non-ribosomally synthesis of the virulent factor lugdunin in *S. lugdunensis* [24], was less abundant under iron limitation condition. This is in agreement with the observation that *S. lugdunensis* IVK28 was unable to produce this antibacterial substance under iron-limiting conditions in liquid culture [24]. Taken altogether, the reduced expression of these polypeptides may, at least in part, explain the decreased virulence of *S. lugdunensis* incubated with DIP observed in *G. mellonella* model of infection. This animal model is a pertinent tool to study the virulence and pathogenesis of a wide range of microorganisms including *S. lugdunensis* [25, 26]. The larvae of *G. mellonella* have an innate immune system which makes possible to obtain relevant information on the infection process, the breeding is easy and inexpensive and the injected doses of bacterial can be defined. In addition, the larvae can withstand at 37 °C which favors the study of pathogenic bacteria growing at this temperature [26]. Our results revealed that the presence of iron was important for its pathogenicity because infection with bacterial cells pre-incubated in BHI supplemented with DIP significantly increased the worms’ survival. Because the iron limitation response led to a reduced ability to cope with oxidative stress, bacteria would become more susceptible to phagocytosis in the hemolymph of caterpillar so less virulent.

## Conclusions

We identified 222 proteins more and 127 less abundant in *S. lugdunensis* incubated under iron-limited condition leading to characterization of the first stress proteome in this species. Based on these data, further phenotypical studies revealed that iron played a dual role for *S. lugdunensis*: a low iron content promoted biofilm formation, which may be in favor of colonization, whereas these ions were required for virulence and to cope with H_2_O_2_. In this context, our global analysis strongly suggests that enzymes involved in the oxidative stress response could play a key role in the ability to persist in the host.

## Methods

### Bacterial cell and growth conditions

The sequenced *S. lugdunensis* N920143 strain was used in this study (GenBank FR870271) [4]. Overnight cultures of *S. lugdunensis* were diluted 2:100 in Brain Heart Infusion (BHI) without or with 350 μM 2,2′-dipyridyl (DIP) (Sigma-Aldrich, Saint Louis, Mo, USA) as previously described [2, 27]. For complementation experiments, 2 mM FeSO_4_ were added. To test the oxidative stress response, 0.4 mM H_2_O_2_ was added. These bacterial suspensions were used to fill the wells of a 96-wells flat-bottom sterile polystyrene microplates. Growth measurements (OD at 600 nm, every 10 min) were performed using the microtiter plate reader Tecan infinite 200 pro (Tecan, Männedorf, Switzerland) and *p*-values were determined using a variance Student t-test.

### Transmission electron microscopy

10 ml of bacterial cell cultures at the onset of stationary phase (OD of 1) and after 24 h in BHI without or with 350 μM DIP were centrifuged (5000 x g) for 10 min at 4 °C. Cell pellets were washed twine with saline buffer and then 800 μl of glutaraldehyde solution 1% were added for fixing. Bacteria were visualized on 200-mesh nickel EM grids (mesh diameter of 74 μm) (Leica Microsystems, Wetzlar, Germany), coated with 2% Formvar (a polyvinyl formal resin) (Monsanto Chemical Company, St. Louis, MI, USA). All observations were performed at CMABio, the Center for Microscopy Applied to Biology (Caen, France) of Normandy University. Observations were performed on JEO1 1011 transmission electron microscopes operating at 80 kV. Images were acquired with a Gatan Orius 200 camera, and processed with Gatan Digital Micrograph software (Gatan, Pleasanton, CA, USA).

### Biofilm formation

Overnight cultures of *S. lugdunensis* were diluted to obtain OD of 0.1 in BHI alone or with 350 μM DIP. Sterile polystyrene microplates were loaded with 100 μL of bacterial suspensions and incubated for 24 h at 37 °C. Biofilm formation was detected using the method described by Christensen et al. [28]. Adherent cells were stained with 0.1% crystal violet for 15 min and, after three washings, wells were air dried. For quantitative estimation of the biofilm density, bound crystal violet was solubilized with 70% ethanol and the absorbance of the solubilized dye was read at 600 nm. Three independent experiments (each in duplicate) were performed and *p*-values were determined using a two-tailed, two-sample unequal variance Student t-test using GraphPad.

### H_2_O_2_ killing assays

Resistance of *S. lugdunensis* N920143 to oxidative killing by H_2_O_2_ was tested as described by Verneuil et al. with slight modifications [29]. Bacteria were grown 24 h in BHI broth and sub-cultured in 10 ml BHI, BHI with 350 μM DIP or BHI with DIP and 2 mM FeSO_4_ broth at a starting density of OD600 at 0.1. Cultures were grown to mid-exponential phase (OD600  =  0.5), final concentration of 1 mM H_2_O_2_ was added, placed into a 37 °C water bath. Samples were taken immediately, 1 h and 2 h following H_2_O_2_ challenge, and rapidly diluted in 0.9% NaCl. Viability was determined by spreading of appropriate serial dilutions on BHI agar and colony forming units (CFU) were determined after 24 h incubation at 37 °C.

### Infection experiments

Infection of *G. mellonella* larvae with *S. lugdunensis* was performed as previously described by Lebeurre et al. [26]. Larvae were infected subcutaneously with 10 μl of cells suspensions of *S. lugdunensis* (around 1 × 10^7^ CFU) from an overnight culture in BHI, BHI with 350 μM DIP or BHI with DIP and 2 mM FeSO_4_. Prior the injection, bacteria were washed and adjusted at the same concentration in saline buffer to avoid presence of DIP into the host and CFU were counted on agar plates after serial dilutions. For each test, ten insects were infected and the experiments were repeated at least four times. As control, sterile saline buffer were injected to larvae. Larval mortalities were then monitored at one, two and 3 days post-infection. Results were analyzed using a one-way analysis of variance with a Bonferroni correction following “R” packages. For all comparisons, a *p*-value less than 0.05 was considered as significant.

### Mass-spectrometry analysis, peptide sequencing and protein precursor identification

In order to extract total proteins, 200 ml of bacterial cell culture were centrifuged (6000 x g) for 15 min at 4 °C. Cell pellets were washed twice with recovery buffer (Tris HCl 50 mM, Na_2_SO_4_ 50 mM, glycerol 15%) and incubated for 12 h at − 80 °C. Cell pellets were transferred into screw top tubes containing 500 μl of glass beads. Cells were disrupted using the Fast Prep instrument (MP Biomedical LLC, Santa Ana, CA, USA) for 3 min at 6.5 m/s. The lysate was centrifuged for 10 min at 10,000 g at 4 °C to remove the cell debris and the supernatant was transferred into a new tube. Proteins were prepared from three independent biological replicates. The protein dosage was realised using the “Pierce BCA protein assay kit” (Thermofisher, Waltham, MA, USA).

Five μg of each protein extract were first prepared using a modified GASP protocol [30]. Samples were digested with trypsin/Lys-C overnight at 37 °C.

For nano-LC fragmentation, protein or peptide samples were first desalted and concentrated onto a μC18 Omix (Agilent) before analysis. The chromatography step was performed on a NanoElute (Bruker Daltonics, Billerica, MA, USA) ultra-high pressure nano flow chromatography system.

MS experiments were carried out on a TIMS-TOF pro mass spectrometer (Bruker Daltonics) with a modified nano electrospray ion source (CaptiveSpray, Bruker Daltonics). A 1400 spray voltage with a capillary temperature of 180 °C was typically employed for ionizing. MS spectra were acquired in the positive mode in the mass range from 100 to 1700 m/z. In the experiments described here, the mass spectrometer was operated in PASEF mode with exclusion of single charged peptides. A number of 10 PASEF MS/MS scans was performed during 1.25 s from charge range 2–5.

Mass spectrometry raw files were processed with MaxQuant version 1.6.7.0. MS/MS spectra were searched by the Andromeda search engine against the Uniprot *S. lugdunensis* database. A maximum of two missing cleavages were allowed, the required minimum peptide sequence length was 7 amino acids, and the peptide mass was limited to a maximum of 4600 Da.

Decoy database hits, proteins identified as potential contaminants, and proteins identified exclusively by one site modification were excluded from further analysis. Label-free protein quantification was performed with the MaxLFQ algorithm that allows accurate proteome-wide label-free quantification by delayed normalization and maximal peptide ratio extraction requiring a minimum ratio count of 1 [31]. All other MaxQuant parameters were kept at their default values. Bioinformatic analysis and visualization was performed in Perseus. Two sample tests were performed using Student’s T test with a Permutation-based FDR of 0.05.

### RNA extraction and RT-qPCR

RNAs were extracted from cells harvested at late-exponential phase and at the same OD of 1. Cells pellets were incubated for 12 h at − 80 °C. RNAs were extracted using the “Direct-zol RNA miniprep kit” (Zymo Research, Irvine, CA, USA). Genomic contaminations were removed by treatment with Turbo DNase according to the manufacturer recommendations (ThermoFisher). For cDNA synthesis, 1 μg of RNAs was reverse transcribed with the “BIORAD iScript Select cDNA synthesis Kit” kit (Bio-Rad, Hercule, CA, USA). For each condition, RT-qPCR experiments have been carried out using three independent RNA samples. Primers (5′ to 3′) are listed in Table S2. For gene expressions, transcript levels were determined by the DeltaDelta Ct method using the *adk* gene as a housekeeping control gene. The Student t-test was used to determine the statistical significance between the samples. For all comparisons, fold changes (FC) > 2 or < − 2 with a *p*-value less than 0.05 were considered as significant.

## Supplementary information

**Additional file 1: Table S1.** List of proteins significantly upregulated and downregulated of growing cell (OD of 1) of *S. lugdunensis* N920143 incubated in BHI with 350 μM DIP compared to grown in BHI.

**Additional file 2: Table S2.** List of oligonucleotides used in this study.

**Additional file 3: Figure S1.** Electron microscopy photographs of *S. lugdunensis* cells. A-C : Bacteria cultivated in BHI until OD of 1 (A) and during 24h (C). B-D : Bacteria cultivated in BHI with 350 μM DIP until OD of 1 (B) and during 24h (D). No significant difference in morphology and cell wall thickness was observed.

**Additional file 4: Figure S2.** Representative growth curves of *S. lugdunensis* N920143 in BHI (continuous line), in BHI with 350 μM DIP (hatched continuous line), in BHI with 0.4 mM H_2_O_2_ (spaced dashed line) and in BHI with 350 μM DIP and 0.4 mM H_2_O_2_ (tight dashed line).

## Data Availability

The LC-MS/MS proteomics data have been deposited to the ProteomeXchange Consortium via the PRIDE [32] partner repository with the dataset identifier PXD021832 (https://www.ebi.ac.uk/pride).

## Abbreviations

DIP: 2,2′-dipyridyl
CoNS: Coagulase-negative *Staphylococcus*
Isd: Iron-regulated surface determinant.
PMNs: Polymorphonuclear neutrophils
RNS: Reactive nitrogen species
BHI: Brain Heart Infusion
