# Supplementary figures and images for: Phenotypic and proteomic approaches of the response to iron-limited condition in Staphylococcus lugdunensis
Source: BMC Microbiol. 2020 Oct 28;20:328. doi: 10.1186/s12866-020-02016-x (PMC7594282; doi:10.1186/s12866-020-02016-x)

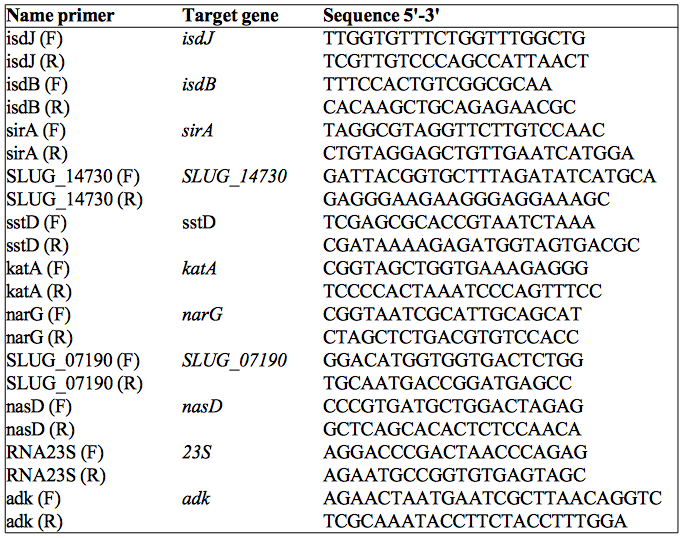

Supplement: Supplementary file 2 — Additional file 2: Table S2. List of oligonucleotides used in this study. [file 12866_2020_2016_MOESM2_ESM.png]

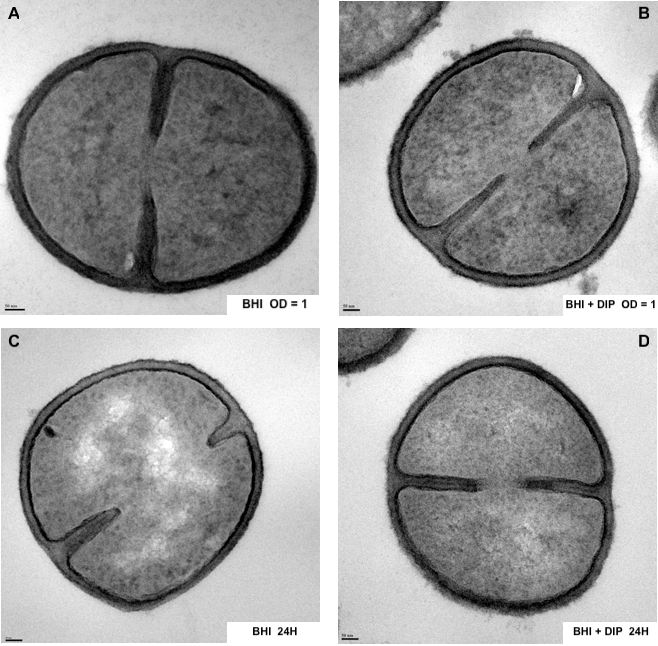

Supplement: Supplementary file 3 — Additional file 3: Figure S1. Electron microscopy photographs of S. lugdunensis cells. A-C : Bacteria cultivated in BHI until OD of 1 (A) and during 24h (C). B-D : Bacteria cultivated in BHI with 350 μM DIP until OD of 1 (B) and during 24h (D). No significant difference in morphology and cell wall thickness was observed. [file 12866_2020_2016_MOESM3_ESM.png]

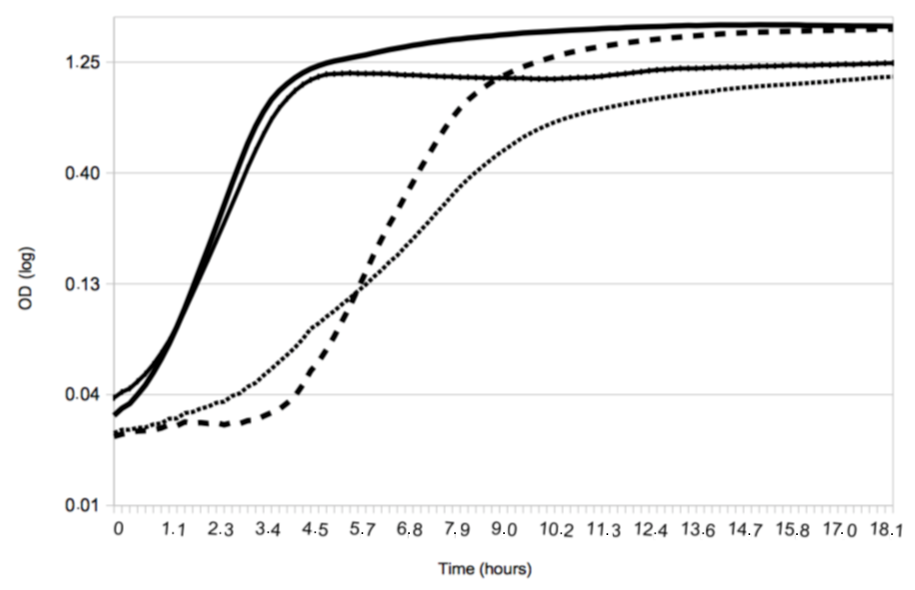

Supplement: Supplementary file 4 — Additional file 4: Figure S2. Representative growth curves of S. lugdunensis N920143 in BHI (continuous line), in BHI with 350 μM DIP (hatched continuous line), in BHI with 0.4 mM H2O2 (spaced dashed line) and in BHI with 350 μM DIP and 0.4 mM H2O2 (tight dashed line). [file 12866_2020_2016_MOESM4_ESM.png]
